# Effect of Steel Slag Aggregate on Pavement and Flame-Retardant Performance of Warm-Mixed Flame-Retardant Asphalt Concrete

**DOI:** 10.3390/ma14030635

**Published:** 2021-01-29

**Authors:** Yanfei Ren, Meizhu Chen, Tianyuan Yang, Shaopeng Wu, Kaifeng Wang

**Affiliations:** 1 State Key Laboratory of Silicate Materials for Architectures, Wuhan University of Technology, Wuhan 430070, China; 234505@whut.edu.cn (Y.R.); chenmzh@whut.edu.cn (M.C.); yangty2017@whut.edu.cn (T.Y.); wusp@whut.edu.cn (S.W.); 2 School of Transportation, Wuhan University of Technology, Wuhan 430070, China

**Keywords:** warm-mixed asphalt concrete, flame retardant, steel slag, pavement performance

## Abstract

With the rapid development of tunnel construction, tunnel safety and the shortage of high-quality aggregates have concerned researchers so that this issue has become a research hot spot in the past few years. In the present study, we intended to prepare warm-mixed flame-retardant asphalt concrete using steel slag aggregate and evaluate its pavement and flame retardant performance. In this regard, the chemical composition and microstructure of the steel slag were studied using X-ray fluorescence analysis (XRF) and scanning electron microscopy (SEM). Then diverse pavement performances, including the dynamic stability, immersion Marshall, freeze–thaw splitting strength and low-temperature bending, were investigated for the warm-mixed flame-retardant asphalt concrete with steel slag aggregate. Moreover, a creative method of the flame spray gun combustion test was proposed to characterize the combustion degree and evaluate the flame-retardant performance of the asphalt concrete with steel slag. The experimental results show that the high-temperature and moisture stability performance are improved due to the addition of steel slag, however, the low-temperature performance is reduced for the warm-mixed flame-retardant asphalt concrete while it is still higher than the requirement value of the Chinese specification (GB/T 30596-2014). Meanwhile, the ignition temperature is increased and the ignition time is delayed for warm-mixed flame-retardant asphalt concrete because of the addition of steel slag. It is concluded that asphalt concrete with steel slag has excellent flame-retardant performance so that it is an appropriate choice for tunnel pavement.

## 1. Introduction

Asphalt pavements are the mainstream pavements in the world due to their short construction period, convenient maintenance, and comfortable driving [1,2,3], and consequently, they have been widely used in tunnel engineering. However, volatile organic compounds (VOCs) emission of traditional hot-mixed asphalt concrete is large during construction, while the semi-closed environment and poor ventilation of tunnels make VOCs pollution more prominent. Meanwhile, asphalt is a kind of inflammable material and will lead to the burning of asphalt pavement when there is a fire accident in the tunnel, which increases the difficulty of rescue and produces a large amount of VOCs, causing more serious pollution and endangering people’s lives [4,5]. In order to resolve this shortcoming, warm-mixed flame-retardant technology has been widely studied in the past few years [6]. Studies show that this technology can reduce the construction temperature of asphalt concrete and increase the safety performance of a pavement [7,8].

Recently, many scholars have studied warm-mixed asphalt pavement and flame-retardant asphalt pavement technologies [9,10]. In particular, Zhao Jiang et al. [11] studied the influence of Sasobit on the warm-mixed asphalt and its mixture performance. They found that Sasobit significantly reduces the asphalt viscosity at high temperatures and improves the high-temperature performance of an asphalt mixture. Moreover, Huang Zhiyi et al. [12,13] studied the combustion of tunnel asphalt pavement. They used gasoline to burn asphalt concrete and investigated the flame-retardant performance of asphalt concrete for different parameters, including flame size, combustion time and sample integrity after combustion. Zhu Zuhuang [14] studied the preparation and performance of warm-mixed flame-retardant asphalt. They showed that the prepared warm-mixed agent and flame retardant improve the high-temperature performance of asphalt, thereby significantly improving the flame-retardant performance of asphalt.

Chen Jinmei [15] studied the performance of the flame-retardant asphalt mixture and found that this mixture increases the retained Marshall stability (*MS_0_*) and tensile strength ratio (*TSR*) of the asphalt concrete by about 7%. Further investigations showed that the bending stiffness modulus of the asphalt concrete at the low temperatures reduces by about 20%, while the corresponding dynamic stability increases by 17%. Qiao Jiangang et al. [16] evaluated the flame-retardant effect of the warm-mixed flame-retardant mixture using the limiting oxygen index (LOI), combustion quality loss and residual road performance. Accordingly, they found that there exists a synergistic effect of warm-mixed agent and flame retardant, which improves the flame-retardant effect of the warm-mixed flame-retardant mixture. However, Liu Xin [17] found that warm-mixed agent and flame retardant suppress the flame-retardant performance for rubber-modified asphalt mixture, and consequently the amount of these substances in the mixture should be controlled. Li Ruixia et al. [18] found that Sasobit, as a warm-mixed agent, is not conducive to flame retardant performance, because it increases the asphalt viscosity.

Chinese tunnel construction has developed rapidly in the past few decades. Currently, China is known as the country with the largest number of tunnels and the longest tunnels in the world. On the other hand, the construction and maintenance of tunnel asphalt pavement require a considerable amount of high-quality natural aggregates. This issue not only imposes a high cost but also damages the ecological environment. As an innovative solution, researchers have applied steel slag to the asphalt concrete. Jiao Wenxiu et al. [19] found that the thermal conductivity of asphalt concrete increases when steel slag replaces 50 wt.% (weight%) of diabase, while it decreases for 100% steel slag. Moreover, Ma Lili et al. [20] proposed a surface waterproof structure to restrain the volume expansion of steel slag and improved the pavement performance of steel slag asphalt mixture, accordingly. Chen Zongwu et al. [21] studied the water stability performance of steel slag asphalt concrete. They found that weathered and silicone surface modification can improve the water stability of steel slag asphalt mixture.

Extensive researches indicate that the addition of the steel slag improves the performance of asphalt concrete in wet and high-temperature environments. Meanwhile, it resolves the problem of resource waste and environmental pollution caused by the long-term accumulation of steel slag. S.A. Ziaee et al. [22] investigated the effect of Sasobit additive and partial replacement of coarse aggregates with electric arc furnace steel slag (EAFS) on the mechanical properties of hot-mixed asphalt (HMA) and warm-mixed asphalt (WMA) mixtures. They found that the optimum amount of steel slag to improve the mechanical properties of the mixtures is about 50 wt.%. However, steel slag is rarely applied in the warm-mixed flame retardant asphalt concrete.

The main purpose of the present article is to prepare warm-mixed flame-retardant asphalt concrete with steel slag and study the influence of the steel slag aggregate on the flame retardant and pavement performances of the warm-mixed asphalt concrete. Since steel slag has reasonable thermal properties, high specific heat capacity and low thermal conductivity, it has the potential to improve the flame-retardant performance of asphalt pavement. In this regard, the chemical composition and surface structure of the steel slag were studied by X-ray fluorescence analysis (XRF) and scanning electron microscope (SEM). This article is expected to provide a theoretical guideline for investigating the influence of steel slag on the characteristics of the asphalt concrete. Meanwhile, an experiment was designed to evaluate the flame-retardant performance of the prepared asphalt concrete.

## 2. Materials and Methods

### 2.1. Materials

#### 2.1.1. Asphalt

Studies show that styrene butadiene styrene (SBS) modified asphalt has superior durability and high-temperature stability compared with ordinary petroleum asphalt [23]. Accordingly, it has been widely applied in pavement engineering. In the present study, SBS modified asphalt is used to prepare asphalt concrete. In order to determine the basic properties of the SBS modified asphalt, softening point, ductility, and penetration tests were conducted with respect to Standard Test Methods of Bitumen and Bituminous Mixtures for Highway Engineering (JTG E20-2011). The basic properties of the SBS modified asphalt are presented in Table 1.

#### 2.1.2. Warm-Mixed Asphalt (WMA) Additive

Sasobit (Chongqing Pengfang Transportation Technology Company, Chongqing, China) was used as a WMA additive in this paper. This additive can be added to improve the fluidity of asphalt at the temperature of above 119 °C [24]. The content of Sasobit is 2.0 wt.% of asphalt. The main properties of Sasobit are shown in Table 2.

#### 2.1.3. Flame Retardant

FRMAX (Haichuan company, Shenzhen, China) was used as a flame retardant in this paper. It consists of several organic and inorganic compounds. The flame retardant can decompose when it is heated, thereby absorbing heat and reducing the asphalt temperature and generating nitrogen to prevent combustion [25]. Its basic properties are shown in Table 3. The dosage of this additive was 8.0% by the weight of asphalt.

#### 2.1.4. Aggregates

In the experiments, basalt and steel slag were used as aggregates. They were provided from Wuhan (Wuhan Iron and Steel Company) and Shanghai (China Baowu Steel Group), China, respectively. The main properties of these aggregates are shown in Table 4. All specifications meet the requirements of the Testing Procedures of Aggregate for Highway Engineering (JTG E42-2005) (Inspection and Quarantine of the People’s Republic of China, 2005).

**Figure 1:**
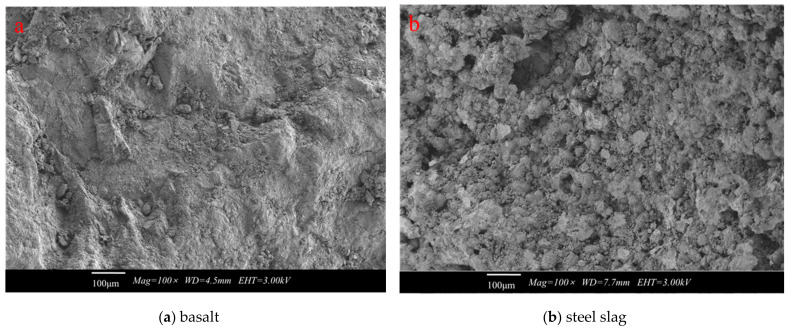
SEM images of different aggregates (2.36–4.75 mm). (**a**) basalt; (**b**) steel slag.

Scanning electron microscope (SEM) is an effective analytical tool to study the surface morphology and structure of aggregate particle. Dry representative samples (2.36–4.75 mm) of basalt and steel slag were glued on the sample tables and plated with gold. SEM images of basalt and steel slag are shown in Figure 1. It is observed that the steel slag has more pores and a larger surface area. This means that the steel slag can absorb more asphalt, which can significantly increase the adhesion between asphalt and aggregates.

X-ray fluorescence analysis (XRF) can be applied to investigate the chemical composition of substance. In this regard, a XRF spectrometer (PANalytical.B.V, Almelo, the Netherlands) was applied in the experiments to determine elements with atomic numbers from 8 to 95. Moreover, XRF was used to characterize the composition and content of oxides in steel slag using a Rh anode tube. Dry representative samples containing difference particle sizes were crushed to obtain XRF samples with particles passing through the 75 μm sieve. XRF of the steel slag is shown in Table 5. It is observed that compared with basalt, steel slag contains more Ca and Fe, but less Si. This indicates that the basicity of steel slag is higher than that of basalt. Further investigations show that asphalt is an acidic material, which has good adhesion with alkaline aggregates such as limestone and basalt [26,27]. Steel slag has high basicity, which means that the adhesion between steel slag and asphalt is excellent.

To sum up the foregoing discussions, steel slag has high potential as an alternative aggregate in the asphalt concrete. Steel slag has a similar chemical composition to basalt and has a rough and porous surface structure.

#### 2.1.5. Design of Aggregate Gradation

**Figure 2:**
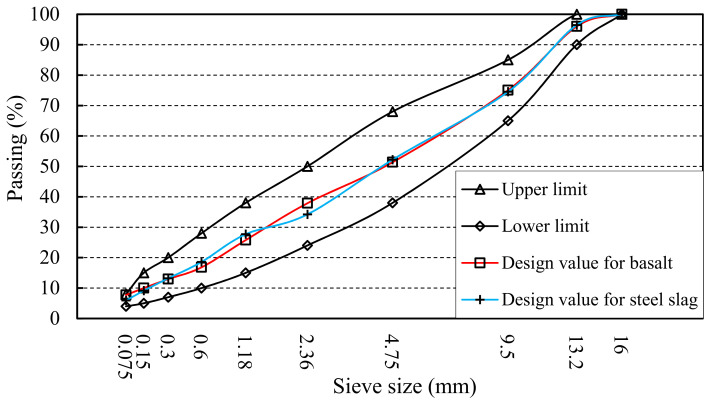
Aggregate gradation curve of AC-13.

The aggregate gradation of AC-13, which was used in the paper, was designed according to the ideas of the Super-pave system, and the optimum asphalt content was determined by Marshall Test method [28,29,30]. The design results of the aggregate gradation are shown in Figure 2. Moreover, composition and preparing temperature of different samples are shown in Table 6. It is worth noting that the steel slag aggregate gradation refers to 100% steel slag, including 0–2.36 mm aggregate.

### 2.2. Experimental Methods

#### 2.2.1. Pavement Performance Tests

##### High-Temperature Performance

**Figure 3:**
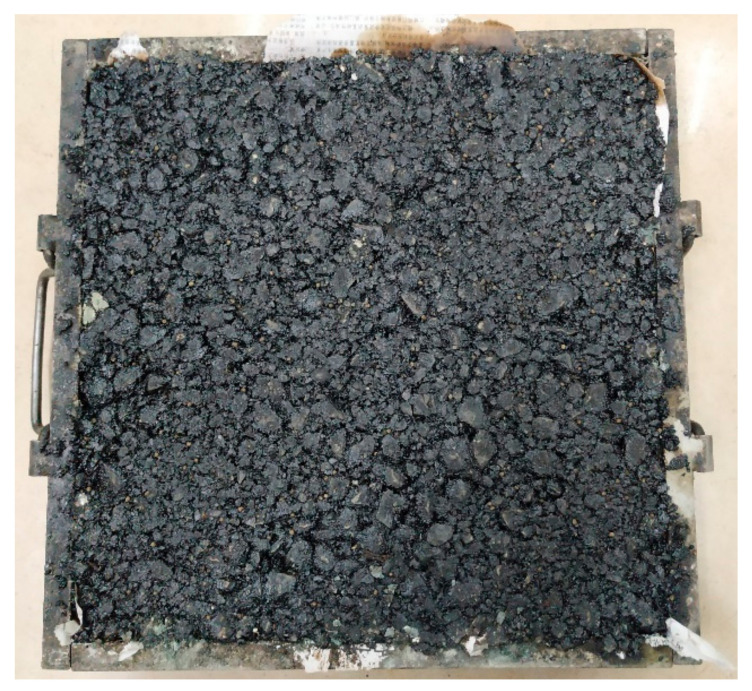
Standard rutting plate specimen.

The wheel tracking test was selected to evaluate the high-temperature performance of the asphalt concrete according to Standard Test Methods of Bitumen and Bituminous Mixtures for Highway Engineering (JTG E20-2011). To this end, standard rutting plate specimens (Figure 3) with dimensions of 300 mm × 300 mm × 50 mm were prepared. The dynamic stability (DS) was tested after maintaining the specimens for 5 h at 60 °C.

##### Moisture Stability

Immersion Marshall and freeze–thaw indirect tensile tests were utilized to evaluate the moisture stability of the asphalt concrete according to Standard Test Methods of Bitumen and Bituminous Mixtures for Highway Engineering (JTG E20-2011). The immersion Marshall test is categorized into two groups, including the control group, and the experimental group. The Marshall stability (*S*_1_) was tested after immersion in a 60 °C water bath for 0.5 h. The immersion Marshall stability (*S*_2_) was tested after water immersion in a 60 °C water bath for 48 h. The residual stability (*MS*_0_) calculated according to the Equation (1) is used as the index to evaluate the moisture stability of the asphalt concrete.

(1) $${\mathrm{MS}}_{0}=\frac{S_{2}}{S_{1}}\times 100\%$$

**Figure 4:**
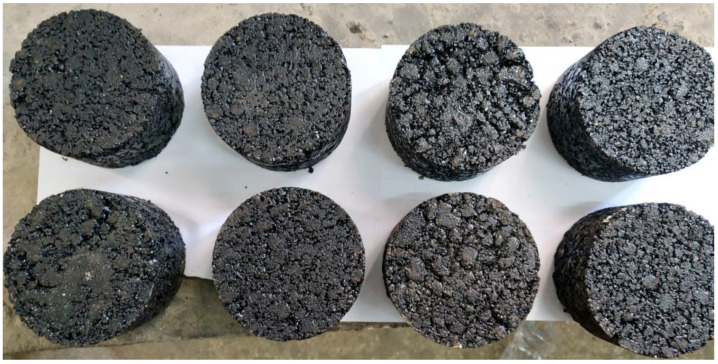
Marshall specimens.

In the freeze–thaw splitting test, Marshall specimens (Figure 4) were divided into two groups, including the control group and the experimental group. The control group was directly immersed in a 25 °C water bath for 2 h, and then the splitting strength (*R*_1_) was tested. The experimental group was soaked in vacuum water for 20 min, then frozen in −18 °C for 16 h, and then placed in a 60 °C water bath for 24 h to complete a freeze–thaw cycle. Then, it was immersed in 25 °C water for 2 h to test its splitting strength (*R_2_*). According to the Equation (2), the residual strength ratio (*TSR*) is calculated.

(2) $$\mathrm{TSR}=\frac{R_{2}}{R_{1}}\times 100\%$$

##### Low-Temperature Performance

**Figure 5:**
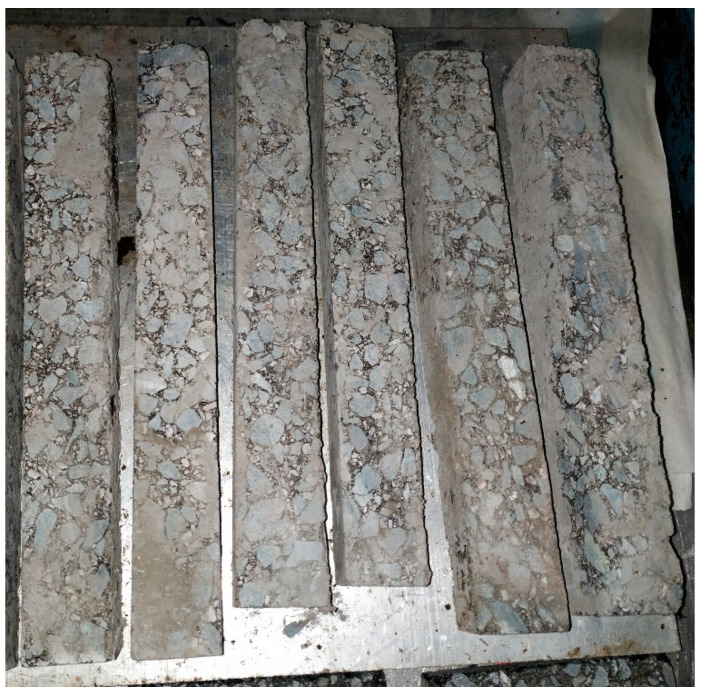
The beam specimens for the low-temperature bending test.

In this section, the low-temperature bending test was utilized to evaluate the low-temperature performance of the asphalt concrete according to Standard Test Methods of Bitumen and Bituminous Mixtures for Highway Engineering (JTG E20-2011). Length×width×height of beam specimens (Figure 5) in the low-temperature bending test was set to 250 mm × 30 mm × 35 mm, respectively. Moreover, the span is set to 200 mm. The specimens were kept at −10 °C for 45 min and then the concentrated load was applied on the mid-span of the specimens with a loading rate of 50 mm/min. The maximum load was determined when a tested specimen was destroyed. According to the Equation (3), the maximum bending tensile strain ($\varepsilon_{B}$) was calculated.

(3) $$\varepsilon_{B}=\frac{6\times h\times d}{L^{2}}$$

where $\varepsilon_{B}$ is the maximum bending tensile strain when the specimen damages (flexural-tensile strain). Moreover, *h*, *L* and *d* denote the height of the mid-span section, the span length of the specimen, and the mid-span deflection when the specimen damages, respectively. It is worth noting that the unit for the abovementioned parameters is mm.

#### 2.2.2. Combustion Test of Loose Asphalt Mixture

**Figure 6:**
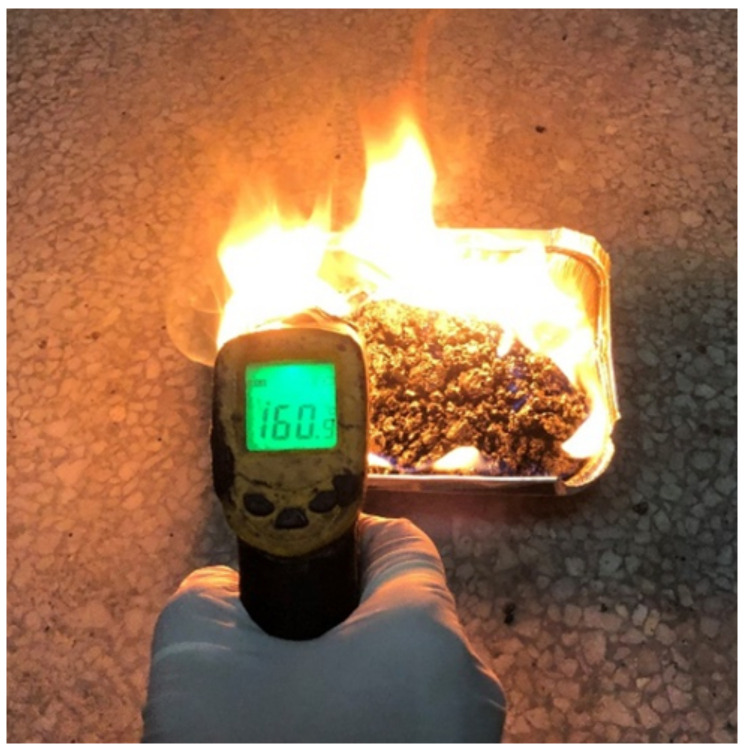
Combustion of the loose asphalt mixture using gasoline.

The loose asphalt mixture is put into a tin foil box and burned with gasoline. It should be indicated that each sample is ignited with a long steel tube [31,32,33]. Figure 6 shows the used method. The combustion duration was recorded and the mixture temperature was measured with a handheld infrared temperature gun (Deli group, Ningbo, China). The amounts of each sample and gasoline were 450 g and 30 mL, respectively.

#### 2.2.3. Combustion Test of the Asphalt Concrete with the Flame Spray Gun

**Figure 7:**
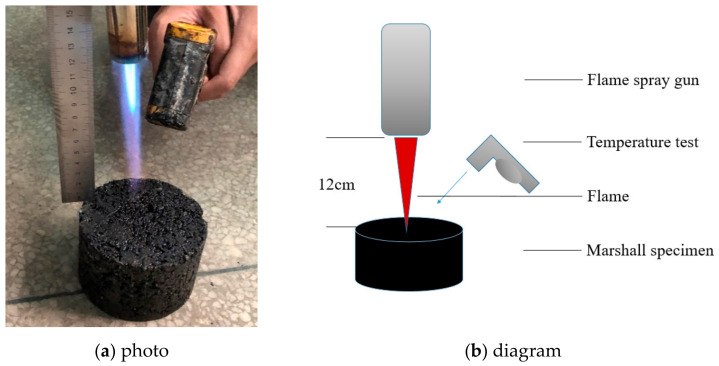
Combustion of asphalt concrete using the flame spraying gun. (**a**) photo; (**b**) diagram.

In this section, the flame spray gun heating method was used to evaluate the burning difficulty of the asphalt concrete. Figure 7 illustrates the applied method. The center of the Marshall specimen was vertically heated by a constant heated heat of the flame spraying gun. It is worth noting that the fuel of the flame spraying gun is butane. When the flame height is about 12 cm, the flame temperature is about 1300 °C. The experiment was stopped when the Marshall’s surface was ignited. It should be indicated that the temperature of the Marshall surface was recorded every 5 s. The ignition time can be used to characterize the combustion difficulty of the asphalt mixture. The temperature distribution of the Marshall specimen was observed using the infrared thermal image instrument (FLIR T420, FLIR Systems Inc, Wilsonville, OR, USA).

## 3. Results and Discussion

### 3.1. High-Temperature Performance

**Figure 8:**
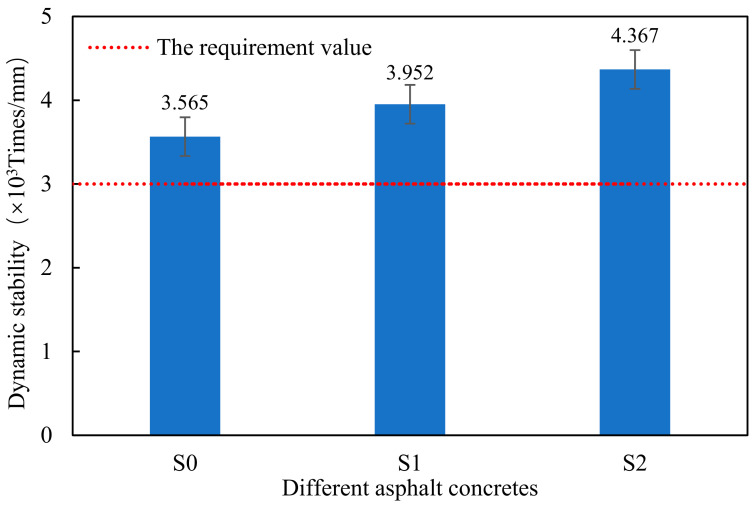
Dynamic stability of different asphalt concretes.

The ability of the asphalt mixture to resist permanent deformation at the high temperature is defined as high-temperature stability. If the high-temperature stability is poor, rutting will appear on the pavement under the action of the driving load, which will reduce the service time of the pavement. The wheel tracking test is a basic test method to evaluate the performance of the asphalt mixture at the high temperatures. The dynamic stability (DS) of the asphalt mixture obtained by the wheel tracking test can reflect the ability to resist rutting at the high temperatures. High DS value means that asphalt concrete has excellent resistance to permanent deformation at the high temperatures. The DS value is mainly related to the type and gradation of the concrete, asphalt content and type, aggregate properties and other factors. Figure 8 shows the DS values of different samples.

It is observed that the DS value of S1 is higher than that of S0, which shows that the warm-mixed flame-retardant asphalt concrete has better performance at the high temperatures. Moreover, it is found that the addition of the steel slag aggregate can increase the DS value of the warm-mixed flame-retardant asphalt concrete. Therefore, the steel slag aggregate can improve the high-temperature performance of the warm-mixed flame-retardant asphalt concrete. The reason for this phenomena is that the steel slag and asphalt have good adhesion. Therefore, the steel slag asphalt concrete has good deformation resistance at the high temperatures.

### 3.2. Moisture Stability Performance

**Figure 9:**
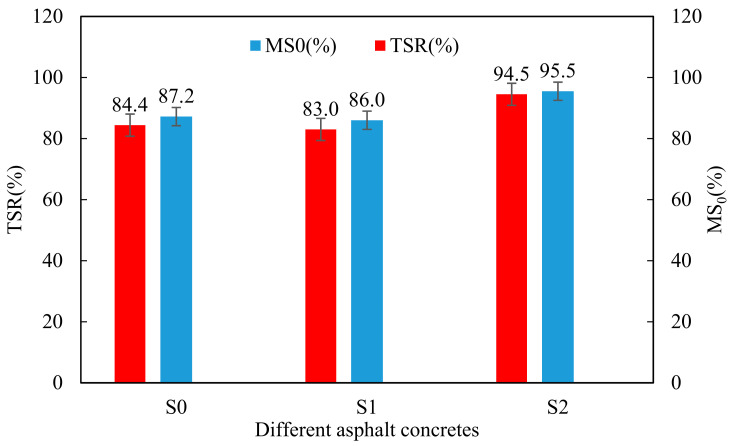
MS_0_ and TSR of different asphalt concretes.

Moisture damage refers to the water entering into the asphalt concrete, which causes the asphalt to peel off from the aggregate surface, resulting in the asphalt pavements damage. Moreover, the moisture stability refers to the influence degree of the asphalt concrete by water, which also shows the water resistance. There are two indices to evaluate the moisture stability. One is the residual Marshall stability (*MS_0_*) obtained by the immersion Marshall test, and the other is the freeze–thaw splitting strength ratio (*TSR*) obtained by the freeze–thaw splitting strength test. High MS and TSR value signify that asphalt concrete has good moisture stability. Figure 9 illustrates the results of *MS*_0_ and *TSR* of different samples.

It is observed that the *MS*_0_ and *TSR* of warm-mixed flame-retardant asphalt concrete are reduced compared with the control sample S0. The test values of *MS*_0_ and *TSR* of S1 are 86.0% (1.2% smaller than S0) and 83.0% (1.4% smaller than S0), respectively. The trend of the experimental phenomenon are consistent with the research results of Li Yingyong [34]. It is found that the addition of the steel slag can significantly improve the *MS*_0_ and *TSR* of the warm-mixed flame retardant asphalt mixture. The test values of *MS*_0_ and *TSR* of S2 are 95.5% (8.3% larger than S0) and 94.5% (10.1% larger than S0), respectively. Steel slag has high basicity and a rough porous surface, which increases the bond strength between the steel slag and asphalt, thereby increasing the mixture capability to resist damage.

### 3.3. Low-Temperature Performance

At the low temperatures, the asphalt becomes brittle and the asphalt mixture is prone to brittle cracking. A low-temperature bending test is a common method to evaluate the low-temperature crack resistance of the asphalt concrete. Generally, high flexural-tensile strain value indicates that asphalt concrete has remarkably good crack resistance at low temperatures. Figure 7 shows the results of the maximum bending tensile strain of different samples.

**Figure 10:**
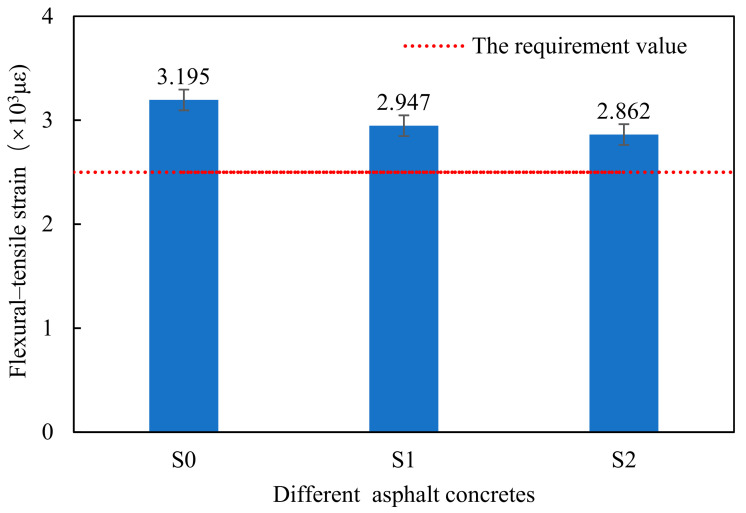
Flexural–tensile strain of different asphalt concretes.

It can be found from Figure 10 that there are different values of flexural-tensile strain for different asphalt concrete. Compared with the control sample S0, the flexural-tensile strain is decreased due to the addition of flame retardant and Sasobit including steel slag aggregate. However, the flexural-tensile strains for S1 and S2 are still higher than the requirement value (2500 με) of the Chinese specification. It can be explained by the fact that the main reason for the asphalt concrete cracking is the brittle fracture of asphalt at the low temperatures. The content of the asphalt used in the warm-mixed flame-retardant asphalt concrete with steel slag is more than that of the samples with basalt aggregate.

### 3.4. Combustion of the Loose Mixture with Gasoline

**Figure 11:**
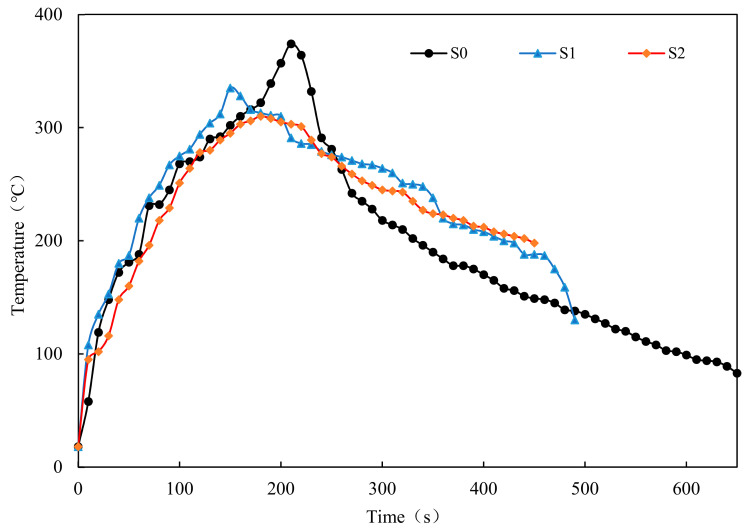
The temperature vs. time of different loose mixtures.

In this section, the combustion test of loose asphalt mixture with gasoline is proposed to evaluate the flame retardant properties of the loose mixture. It should be indicated that the combustion duration and maximum temperature of mixture can be used to characterize its combustion performance. The mixture with good flame retardant performance has short burning time and low maximum temperature. Figure 11 shows the change in the temperature of different loose mixtures with time. Table 7 shows combustion duration and maximum temperature of different loose mixtures.

The results show that the maximum temperature of all samples was about 300–400 °C. The combustion duration of S1 is 160 s shorter than that of S0, and the maximum temperature is reduced by 39 °C. The flame-retardant performance of the warm-mixed flame-retardant asphalt concrete is excellent. The research results of Li Xuelian [35] also get the similar rule. It is worth noting that the combustion duration of S2 is 40 s shorter than that of S1, and the maximum temperature is reduced by 20 °C. The addition of the steel slag improves the flame-retardant performance of the warm-mixed flame-retardant asphalt mixture. When the asphalt is heated above 280 °C, the flame retardant decomposes to absorb the heat and reduce the temperature of the asphalt concrete. Therefore, the flame retardant effect is achieved. The asphalt concrete prepared by the steel slag aggregate has high specific heat capacity and low thermal conductivity [36]. The addition of the steel slag can reduce the combustion temperature of the asphalt concrete and the diffusion of combustion.

### 3.5. Combustion of Asphalt Concrete with the Flame Spray Gun

**Figure 12:**
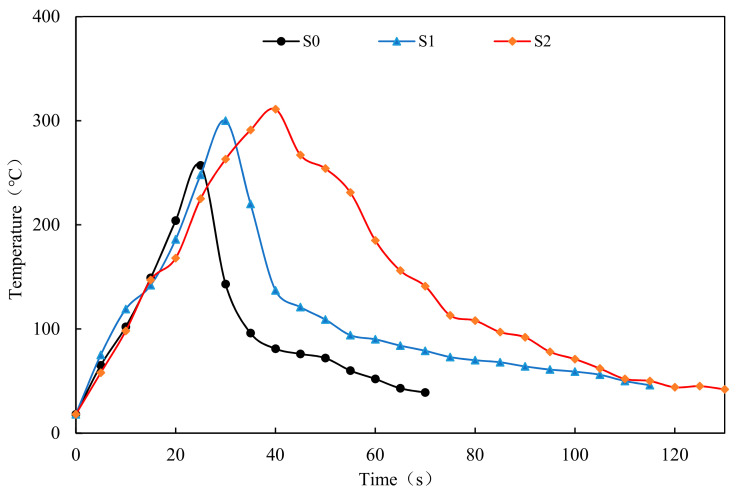
Change in the temperature of different asphalt concrete with time.

In this section, the flame spray gun combustion experiment with Marshall is proposed to evaluate the flame retardant properties of the asphalt concrete. It should be indicated that the longer the ignition time takes and the lower the maximum temperature is, the more difficult the asphalt concrete is to burn. Figure 12 presents the change in temperatures of different asphalt concretes with time. Table 8 shows the duration of ignition and ignition temperature of different samples.

It is found that the ignition temperature of the all samples was about 250–350 °C. The ignition time and the maximum temperature of different asphalt concrete are used to characterize the difference in the flame-retardant performance. The ignition time of S1 is 5 s longer than that of S0, and the maximum temperature increases by 43 °C. It is found that the flame-retardant performance of the warm-mixed flame-retardant asphalt concrete is more reasonable. The ignition duration of S2 is 10 s longer than that of S1, and the maximum temperature increases by 11 °C. The addition of the steel slag improves the flame-retardant performance of the warm-mixed flame-retardant asphalt concrete.

**Figure 13:**
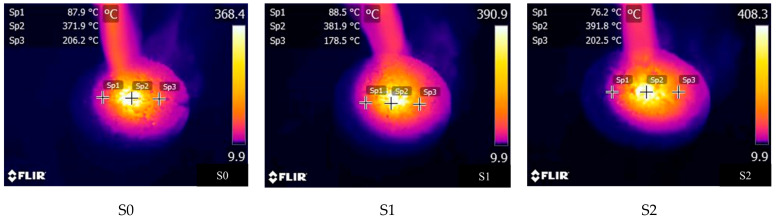
Infrared images of different samples during the ignition process.

Figure 13 shows the infrared images of different asphalt concretes during the ignition process. The temperature in the center of the sample is the highest where it is heated directly by the flame. It is observed that the maximum temperature of Marshall sample was above 360 °C when a small flame appears in the sample. The maximum temperature of the warm-mixed flame-retardant asphalt concrete is 22.5 °C higher than that of the hot-mix asphalt concrete, which indicates that the warm-mixed flame-retardant asphalt concrete has an excellent flame retardant performance. The maximum temperature of the warm-mixed flame-retardant asphalt concrete with steel slag is 17.4 °C higher than that of the warm-mixed flame-retardant asphalt concrete. The addition of the steel slag can improve the flame retardant performance of the warm-mixed flame-retardant asphalt concrete.

## 4. Conclusions

In the present study, the influence of the steel slag on the performance of the warm-mixed flame-retardant asphalt concrete is studied. The following conclusions are drawn from this study.

(1) Steel slag aggregate has high alkalinity and a rough and porous surface. Moreover, it can form strong adhesion with asphalt, which has the potential to increase the pavement performances of the asphalt concrete.

(2) Steel slag can improve the high-temperature performance and moisture stability of the warm-mixed flame-retardant asphalt concrete. However, it reduces the low-temperature performance, which is still higher than the requirement value of the Chinese specification (GB/T 30596-2014).

(3) The addition of steel slag aggregate can improve the flame-retardant performance of the warm-mixed flame-retardant asphalt concrete. The asphalt concrete prepared by the steel slag aggregate has high specific heat capacity and low thermal conductivity. The addition of the steel slag can reduce the combustion temperature of the asphalt concrete and reduce the diffusion of combustion.

## Figures and Tables

**Table 1:** Basic properties of SBS modified asphalt.

Properties		Value	Specifications	Standard
Density (g/cm^3^)		1.032	-	T0603-2011
Penetration (0.1 mm at 25 °C)		53.9	40–60	T0604-2011
Ductility (cm at 5 °C)		42.6	≥20	T0605-2011
Softening point (°C)		78.1	≥75	T0606-2011
Brookfield viscosity (Pa·s at 135 °C)		2.9	≤3	T0625-2011
TFOT residue	Mass loss (%)	0.022	≤0.3	T0610-2011
	Ratio of penetration (%)	67.8	≥65	T0610-2011
	Ductility (cm at 5 °C)	28.4	≥15	T0610-2011

**Table 2:** Characteristics of warm-mixed asphalt additive.

Properties	Values
Appearance	White granules
Density (g/cm^3^ at 25 °C)	0.95
Melting point (°C)	102
Flash point (°C)	290
Brookfield viscosity (Pa·s at 135 °C)	0.012

**Table 3:** Characteristics of the flame retardant.

Properties	Test Value
Appearance	White powder
Effective content (%)	≥85
Density (g/cm^3^ at 25 °C)	2.6
Melting point (°C)	130
Decomposition temperature (°C)	≥280

**Table 4:** Characteristics of aggregates in the asphalt mixture.

Properties		Steel Slag	Basalt	Specifications	Standard
Apparent specific gravity (g/cm^3^)	9.5–16 mm	3.680	2.967	≥2.9	T0304-2005
	4.75–9.5 mm	3.598	2.935	≥2.9	T0304-2005
	2.36–4.75 mm	3.599	2.944	≥2.9	T0304-2005
	0–2.36 mm	3.532	2.979	≥2.9	T0328-2005
Crush value (%)		13.4	9.8	≤20	T0316-2005
Los angeles abrasion (%)		16.9	10.4	≤28	T0317-2005
f-CaO (%)		2.2	-	≤3	YB/T4328-2012

**Table 5:** Chemical composition of steel slag and basalt (%).

Compound	CaO	SiO_2_	Al_2_O_3_	Fe_2_O_3_	MgO	LOI
Steel slag	45.59	18.24	1.43	24.02	6.42	0.67
Basalt	8.43	48.07	19.26	10.46	5.36	3.80

**Table 6:** Composition and preparing temperatures of different asphalt concrete.

Sample	Aggregate	WMA Additive (wt.%)	Flame Retardant (wt.%)	Asphalt Content (wt.%)	Mixing Temperature (°C)	Compaction Temperature (°C)
S0	Basalt	0	0	4.6	180	160
S1	Basalt	2	8	4.6	170	150
S2	Steel slag	2	8	5.4	170	150

Note: S0—Hot mixed asphalt concrete; S1—Warm-mixed flame-retardant asphalt concrete; S2—Warm-mixed flame-retardant asphalt concrete with 100% steel slag.

**Table 7:** Combustion duration and maximum temperature of different loose mixtures.

Type of Mixture	Combustion Duration (s)	Maximum Temperature (°C)
S0	650	374
S1	490	335
S2	450	315

**Table 8:** Duration of ignition and ignition temperature of different samples.

Type of Mixture	Ignition Time (s)	Maximum Temperature (°C)
S0	25	257
S1	30	300
S2	40	311

## Data Availability

Data sharing is not applicable to this article.

## References

[1] Li Q. (2020). Effect of metal hydroxide on flame retardancy and road performance of SBS modified asphalt. Fujian Transp. Sci. Technol..

[2] Xia W. (2020). Combustion Behavior of Bituminours Pavement under Tunnel Fire and Synergistic Inhibition Mechanism of Composite Flame Retardants. Ph.D. Thesis.

[3] Shen A., Su Y., Yang X., Wang H., Zhao W. (2020). Effect of ATH MMT flame retardant on performance of asphalt mixture. J. Chang’an Univ. Nat. Sci. Ed..

[4] Zhang L., Bai Y., Sun W., Zhou Y., Feng J. (2020). Road performance and engineering application of epoxy flame retardant asphalt mixture by post mixing method. Highw. Traffic Technol..

[5] Jin L., Wei J., Fu Q., Zhang Q. (2020). Effect of DBDPE composite flame retardant on properties of SBS asphalt. J. Chang’an Univ. Nat. Sci. Ed..

[6] Wang Y. (2019). Effect of warm mix agent and flame retardant on properties of SBS modified asphalt. Sichuan Build. Mater..

[7] Cao Y. (2018). Research on construction technology of warm mix flame retardant asphalt pavement. Transp. World.

[8] Jia Z. (2020). Application of wsma-13 flame retardant asphalt mastic mixture in extra long tunnel. Transp. World.

[9] Wang Z. (2019). Performance and evaluation of flame retardant asphalt mixture. Shanghai Constr. Technol..

[10] Xiao F., Guo R., Wang J. (2019). Flame retardant and its influence on the performance of asphalt—A review. Constr. Build. Mater..

[11] Zhao J., Pu S., Li W., Chai Z. (2019). Influence of warm mix agent on properties of warm mix asphalt and its mixture. Highway Transp. Technol. Appl. Technol. Ed..

[12] Huang Z. (2007). Investigation of Combustion Mechanism and Safety Experiments of Fire Processes in Long Tunnel with Asphalt Pavement. Ph.D. Thesis.

[13] Huang Z., Wu B., Kang C., Zhu K., Wu K. (2016). Flame retardant and road performance of composite hydroxide modified asphalt. J. Zhejiang Univ. Technol..

[14] Zhu Z. (2011). Research on Preparation and Performance of Warm-Mixed Flame Retardant Asphalt Mixture. Master’s Thesis.

[15] Chen J. (2019). Study on road performance of flame retardant asphalt mixture for tunnel. Western Transp. Technol..

[16] Qiao J., Li W., Guo R., Li Z. (2020). Evaluation of flame retardant effect of warm mix flame retardant asphalt mixture. http://kns.cnki.net/kcms/detail/11.4537.X.20201208.1021.002.html.

[17] Liu X. (2020). Performance and engineering application of warm mix flame retardant rubber composite modified asphalt mixture. Highw. Traffic Guangdong.

[18] Li R., Karki P., Hao P. (2020). Fatigue and self-healing characterization of asphalt composites containing rock asphalts. Constr. Build. Mater..

[19] Jiao W., Sha A., Liu Z., Li W., Jiang W., Qin W., Hu Y. (2020). Study on thermal properties of steel slag asphalt concrete for snow-melting pavement. J. Clean. Prod..

[20] Ma L., Xu D., Wang S., Gu X. (2020). Expansion inhibition of steel slag in asphalt mixture by a surface water isolation structure. Road Mater. Pavement Des..

[21] Chen Z., Jiao Y., Wu S., Tu F. (2018). Moisture-induced damage resistance of asphalt mixture entirely composed of gneiss and steel slag. Constr. Build. Mater..

[22] Ziaee S.A., Behnia K. (2020). Evaluating the effect of electric arc furnace steel slag on dynamic and static mechanical behavior of warm mix asphalt mixtures. J. Clean. Prod..

[23] Hu Y. (2020). Discussion on construction technology of SBS modified asphalt pavement. East. China Highw..

[24] Liu X., Sha A., Li C., Zhang Z., Li H. (2020). Influence of water on warm-modified asphalt: Views from adhesion, morphology and chemical characteristics. Constr. Build. Mater..

[25] Yu X. (2018). Study on technical performance of warm mix flame retardant asphalt concrete. West. Transp. Technol..

[26] Cala S., Caro M., Lleras Y. (2019). Rojas-Agramonte, Impact of the chemical composition of aggregates on the adhesion quality and durability of asphalt-aggregate systems. Constr. Build. Mater..

[27] Xiao Z., Chen M., Wu S., Xie J., Kong D., Qiao Z., Niu C. (2019). Moisture Susceptibility Evaluation of Asphalt Mixtures Containing Steel Slag Powder as Filler. Materials.

[28] Zuo X., Zhai Z. Analysis of mix proportion adjustment of asphalt mixture design. Proceedings of the Academic Exchange Meeting on Construction Technology and Management.

[29] Hua F. (2016). Discussion on mix proportion design of hot mix asphalt mixture. China High. Technol. Enterp..

[30] Zheng C. (2012). Research on warm mix asphalt mixture test and mix proportion design. Highw. Transp. Technol. Appl. Technol. Ed..

[31] Wang X. (2018). Study on the Technology of Flame Retardant Asphalt Mixture in Tunnel. Master’s Thesis.

[32] Zhang K. (2017). Study on Technical Performance of Warm Mixed Flame Retardant Asphalt and Its Mixture. Master’s Thesis.

[33] Dan R. (2016). Research on Application of Warm-Mixed Flame Retardant Asphalt Mixture in Tunnel Pavement. Master’s Thesis.

[34] Li Y., Liu S., Xue Z., Cao W. (2014). Experimental research on combined effects of flame retardant and warm mixture asphalt additive on asphalt binders and bituminous mixtures. Constr. Build. Mater..

[35] Li X., Zhou Z., Deng X., You Z. (2016). Flame Resistance of Asphalt Mixtures with Flame Retardants through a Comprehensive Testing Program. J. Mater. Civil. Eng..

[36] Xu H., Wu S., Li H., Zhao Y., Lv Y. (2020). Study on Recycling of Steel Slags Used as Coarse and Fine Aggregates in Induction Healing Asphalt Concretes. Materials.

